# Atypically presented flank pain in a patient with pulmonary embolism; a case report and literature review of a diagnostic dilemma

**DOI:** 10.1097/MD.0000000000050356

**Published:** 2026-08-28

**Authors:** Mohammed Alra’e, Maaweya Jabareen, Hanan Qunibi, Saleh Juneidi, Wasef Alhroub, Walid Tamimi, Musad Shawar

**Affiliations:** a Faculty of Medicine, Hebron University, Hebron, Palestine; b Radiologist, Faculty of Medicine, Al-Quds University, Hebron, Palestine; c Palestine Red Crescent Society, Hebron, Palestine.

**Keywords:** computed tomography, Deep vein thrombosis, oral contraceptive pills, Pulmonary embolism, venous thromboembolism

## Abstract

**Rationale:**

Pulmonary embolism (PE) is a potentially life-threatening condition with a broad spectrum of clinical presentations, making diagnosis challenging. Although dyspnea, pleuritic chest pain, and cough are common manifestations, atypical presentations may lead to delayed diagnosis and treatment.

**Patient concerns:**

We report the case of a 44-year-old woman who presented with acute right-sided flank pain as her predominant symptom, without evidence of urinary or gastrointestinal pathology.

**Diagnoses:**

Initial abdominal imaging was unremarkable, and laboratory investigations for urinary pathology were negative. Given her risk factors, including recent travel and oral contraceptive pill use, along with subtle chest findings, further evaluation was performed and revealed a right subsegmental PE with minimal pleural effusion.

**Interventions:**

Anticoagulation therapy was promptly initiated.

**Outcomes:**

The patient experienced complete resolution of her flank pain and other symptoms within 72 hours.

**Lessons:**

Abdominal pain associated with PE has been described in the literature; however, isolated flank pain is a rare presentation. Proposed mechanisms include diaphragmatic pleural irritation, pulmonary infarction, and referred pain through shared thoracic sensory pathways. This case highlights the importance of maintaining a high index of suspicion for PE in patients presenting with unexplained atypical pain, particularly when risk factors are present. Early recognition and treatment of atypical PE presentations may prevent diagnostic delays and reduce associated morbidity and mortality.

## 1. Introduction

Venous thromboembolism (VTE), which includes both deep vein thrombosis (DVT) and pulmonary embolism (PE), is a long-term condition that impacts nearly 10 million people globally each year. While significant triggers such as major surgical procedures and active malignancy markedly increase the risk, the majority of VTE cases occur without any identifiable provoking factor.^[1]^

Acute PE represents the most severe form of VTE and is a leading cause of sudden death. Additionally, it is a significant global health concern.^[2]^ In the United States, PE occurs in approximately 500,000–600,000 individuals each year and leads to 200,000–300,000 deaths. It ranks as the third most prevalent cardiovascular condition after acute myocardial infarction and stroke, with an annual incidence of 1–2 cases per 1000 people.^[3]^ In most cases, fatal PE develops as a consequence of deep vein thrombosis (DVT), when a thrombus dislodges and travels to the pulmonary arteries, obstructing the trunk or its branches and leading to the development of potentially fatal obstructive shock and right-sided heart failure.^[2]^

The clinical presentation of PE is highly variable with nonspecific symptoms and can range from mild respiratory symptoms to severe life-threatening obstructive shock. Dyspnea, pleuritic chest pain, cough, hemoptysis, and syncope are typical symptoms of PE, but they are nonspecific.^[2]^ Diagnosis of PE is of utmost importance since early intervention protects from morbidity and mortality.^[4]^

Abdominal pain is a symptom with a huge differential diagnosis, given that abdominal causes can lead to chest pain and vice versa.^[5]^ Flank pain secondary to pulmonary embolism is extremely rare. Herein, we report an atypically presented PE with flank pain, highlighting the necessity of diagnosing and managing such cases as early as possible to avoid delays in the diagnosis of a potentially life-threatening condition.

## 2. Case presentation

A 44-year-old female patient presented to the emergency department (ED) with acute onset flank pain that started 1 day ago. The patient is G4P4A0, with the last conception being 12 years prior to admission. In addition, she had a history of multiple travels by airplane, with the last one being 5 months prior to admission.

She was in her usual state of health until 1 month ago, when she began to develop menstrual irregularities and hot flashes that were controlled by oral contraceptive pills (OCPs). Two weeks later, she developed dyspnea, right-sided chest pain, and an intermittent cough productive of greenish sputum and treated herself with azithromycin without a doctor’s prescription. She claimed that her symptoms improved.

After a few days, the patient started to have gradual-onset, severe right-sided flank pain radiating to the right back, right-sided stitching chest pain that increased with cough and respiration (pleuritic), dyspnea, palpitations, and left calf pain heaviness. The patient denied any history of fever, dysuria, frequency, urgency, nocturia, hematuria, or change in urine color. There was no history of dysphagia, syncope, diaphoresis, nausea, vomiting, or change in bowel habits.

On examination, the patient’s vital signs were a blood pressure of 136/99 mm Hg, a respiratory rate of 22 breath per minute, a heart rate of 100 bpm, a temperature of 36.5°C, and an oxygen saturation of 98%. The abdomen was soft and lax, with no visible abnormalities. The right flank was tender to palpation. There was no suprapubic tenderness. Murphy’s sign and the appendiceal signs were negative as well. The chest examination was significant for the presence of decreased air entry and dullness in the right lower lung. The heart rate was regular, with normal S1 and S2, and no added sounds. Additionally, the left calf was mildly swollen.

Electrocardiogram (ECG) revealed the presence of sinus tachycardia. Labs were significant for mild leukocytosis (12.6 × 10^9^/L) and an elevated C-reactive protein (CRP) of 119 mg/dL. Hemoglobin level (12 g/dL), platelet count (240 × 10^9^/L), urine analysis, creatinine (0.5 mg/dL), and alanine aminotransferase (21 U/L) were normal. Additionally, troponin I was negative as well.

**Figure 1.**
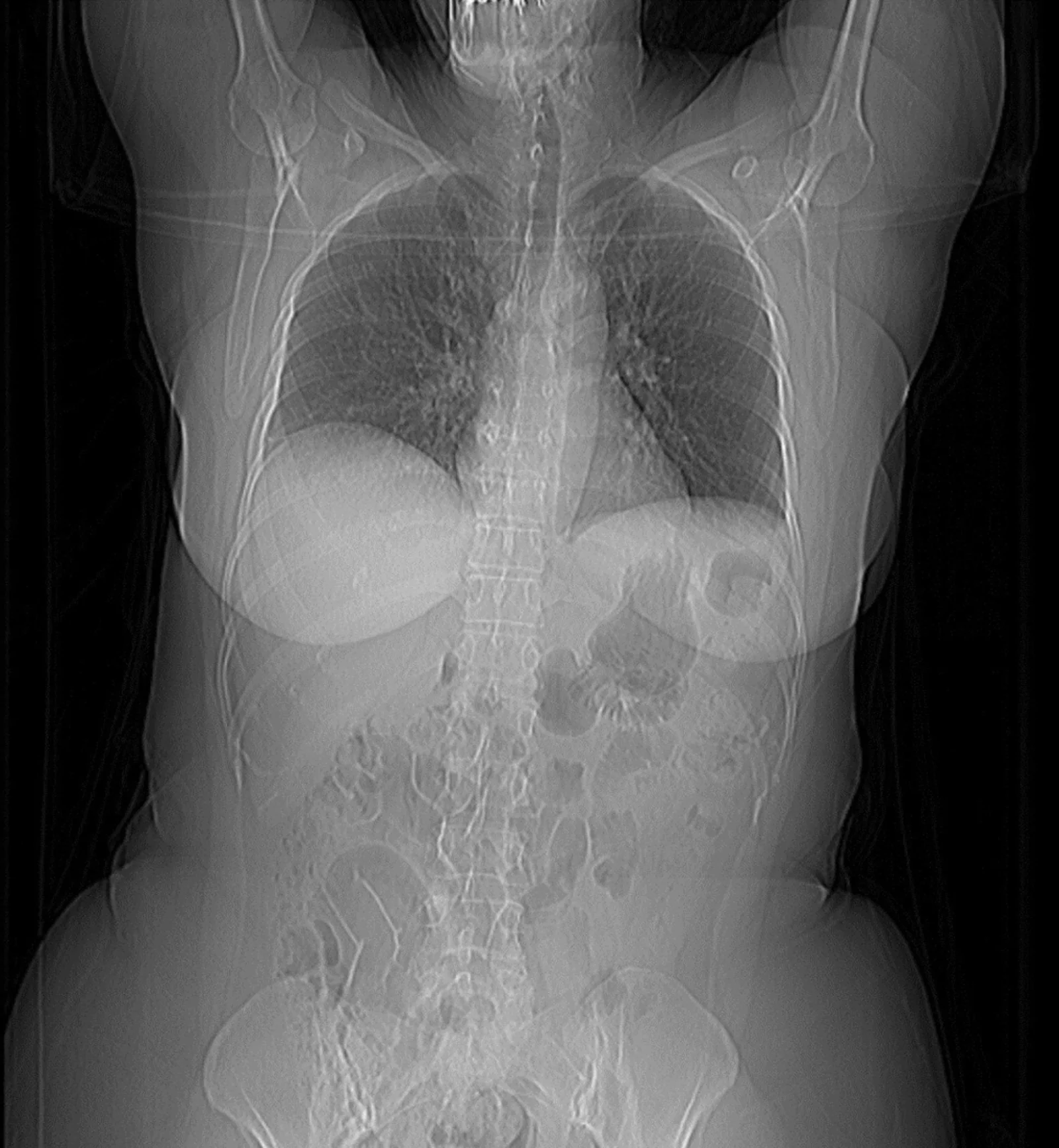
A scout thoracoabdominopelvic x-ray, failing to show any signs of pulmonary embolism or nephrolithiasis. Additionally, the breast shadows mask any potential pulmonary findings behind them.

**Figure 2.**
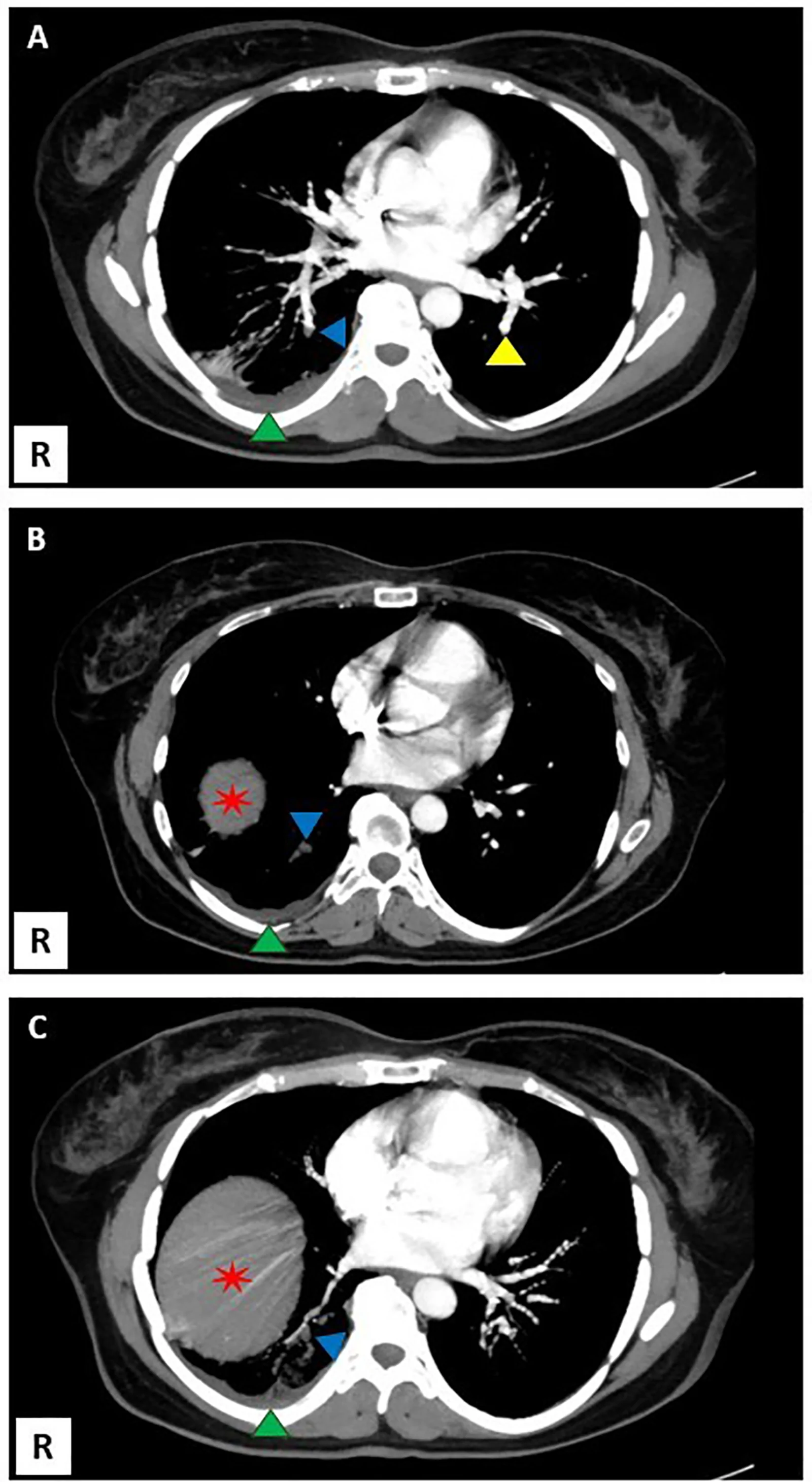
CT angiography at multiple levels, showing 3 sequential slices (from A to C). In A, there is an occlusive pulmonary embolus represented by a filling defect at the right subsegmental branch at which the contrast stopped (blue head arrow). The cutoff flow of the contrast in the vessel is indicated by a hypodense spot (the embolus) versus the normal cut on the left side marked by the yellow head arrow. The green head arrow indicates pleural effusion. The red asterisk indicates an elevated diaphragm, consistent with a collapsed right lung base. R indicates the right side.

**Figure 3.**
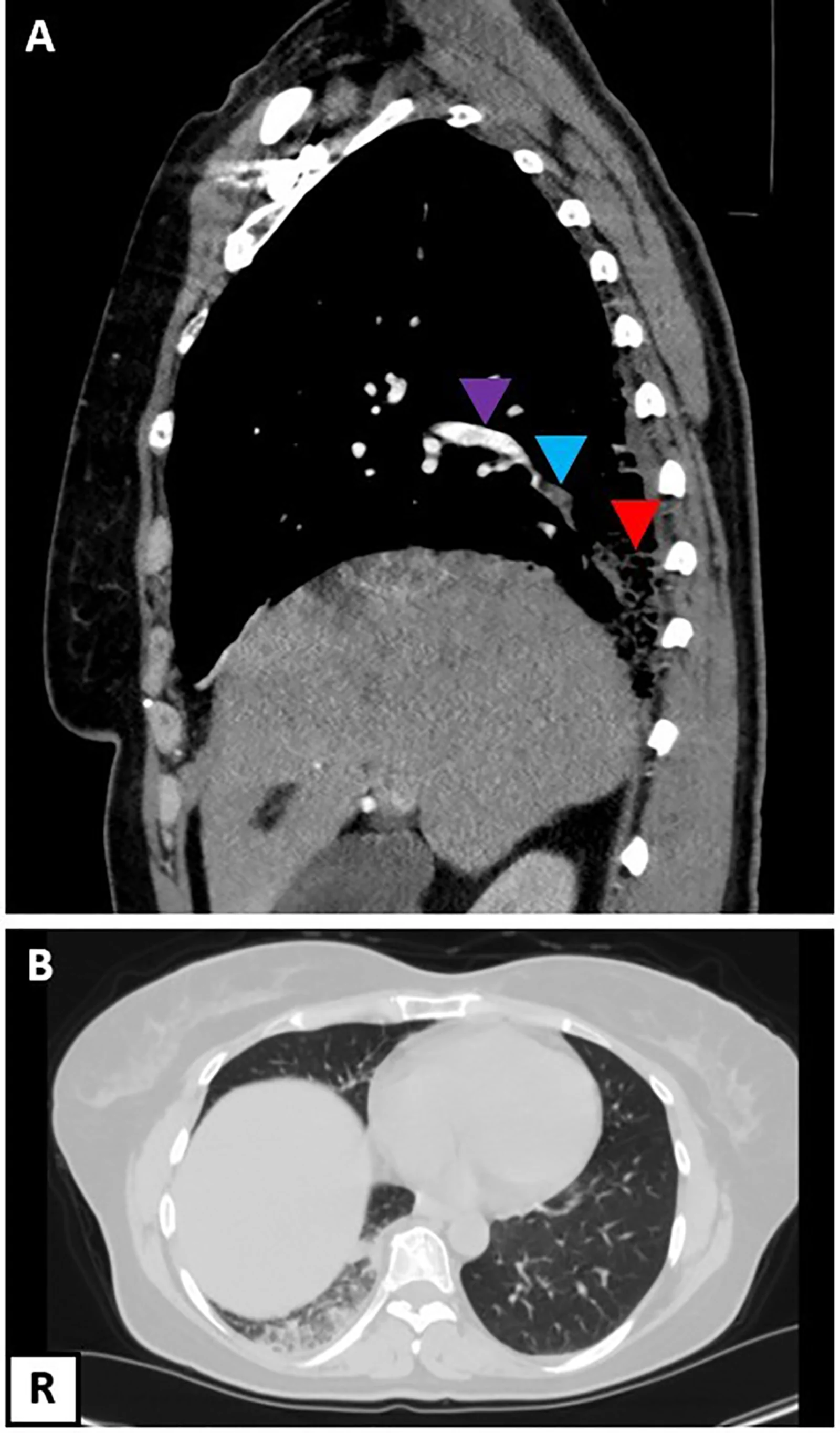
(A) A sagittal view of the right chest showing the obstructing embolus represented as a filling defect (blue head arrow) with proximal dilatation of the right pulmonary artery branch (corresponding to the Palla sign on chest x-ray) marked by the purple head arrow. Additionally, right base collapse is seen (red head arrow). (B) A lung window showing the presence of consolidations at the right lung base, as well as an elevated right diaphragm.

In light of that, and regarding the fact that the flank pain was the top of the patient’s complaints, a urologist was consulted and ordered ultrasonography (US) of the kidneys and abdomen, which was normal and failed to explain the cause of the flank pain. Subsequent abdominopelvic computed tomography (CT) was performed and was normal, with no signs of renal stones, hydronephrosis, perinephric, ureteric, bladder pathology, or other lesions. The liver, gallbladder, and appendix were normal as well. The patient was supposed to have musculoskeletal pain and received paracetamol infusion and a muscle relaxant, but failed to improve symptoms. Further reevaluation of the CT showed an incidentally collapsed lung base. Given the history of OCP intake, recent travel on an airplane, and in light of the mildly swollen left calf and her chest symptoms, PE secondary to provoked DVT was suspected. Prothrombin time (PT) of 12.6 seconds, activated partial thromboplastin time (aPTT) of 25.1 seconds, and international normalization ratio (INR) of 0.97 were within normal limits. However, the D-dimer level was elevated (1101 ng/mL), which prompted subsequent CT angiography of the chest, revealing the presence of right subsegmental PE with minimal pleural effusion (Figs. 1–3). The patient was started on enoxaparin sodium and rivaroxaban, as well as analgesics. Reduction of the CRP (to 59.3 mg/dL) and WBC (to 8.75 × 10^9^/L), improvement of the general condition, and resolution of the presenting flank pain ensued within 72 hours, and the patient was discharged home on oral rivaroxaban for 3 months. Follow-up with lower limb Doppler US was negative for DVT, and anticoagulation was deescalated.

## 3. Discussion

PE is a potentially life-threatening manifestation of VTE, most often resulting from thrombus migration from the deep veins of the lower extremities toward the pulmonary trunk or its branches. The severity depends on where the embolus obstructs. When obstructing the pulmonary trunk (saddle embolus), sudden death can occur. Meanwhile, obstruction of distal branches is less dangerous and can lead to pulmonary infarction, chronic pulmonary hypertension, or even resolution.^[2,6]^

Pulmonary embolism risk factors are either **idiopathic** – such as older age, thrombophilia, obesity, smoking, metabolic syndrome, and long-haul travel – or **provoked**, including immobilization, surgery, trauma, hormonal therapy, pregnancy, cancer, and acute medical illnesses. Virchow’s triad (hypercoagulability, blood stasis, endothelial injury) is a valuable triad that summarizes major mechanisms by which pathological thrombosis occurs. Additionally, infection can trigger thrombosis by unclear mechanisms.^[7,8]^ This underscores that infected patients (such as those with pneumonia) are at higher risk of VTE.

PE is a common yet frequently underdiagnosed condition. A review of literature spanning 1945–2002 found that among 3876 patients in whom PE was identified at autopsy, the diagnosis had been unsuspected or missed before death in 3268 cases (84%).^[9]^ This emphasizes its nonspecific presentation and the need for high clinical suspicion in at-risk patients.

The classical presentation of PE includes symptoms such as dyspnea, pleuritic chest pain, cough, and hemoptysis, with tachypnea and tachycardia being the most commonly observed clinical signs.^[10]^ In addition to the classic symptoms and signs, PE can present with a wide range of other manifestations, such as syncope and alterations in mental status, further complicating its clinical diagnosis.^[11]^

In our case, the primary symptom was flank pain, an exceptionally rare and unusual presentation of pulmonary embolism. According to our review, abdominal pain has been reported in approximately 10–12% of PE cases. In an early report from 1957, abdominal pain was documented in 12.2% of 90 PE patients, with half experiencing it as the chief complaint. A large study published in 2011 similarly identified upper abdominal pain in 10.7% of 1880 PE patients. In recent years, reviews on acute abdomen have increasingly recommended that PE be considered as a differential diagnosis for upper abdominal pain.^[12,13]^ Consequently, the 2015 Japanese guidelines for acute abdomen included acute PE among the critical conditions to be excluded during diagnosis.^[14]^

A study conducted in 2019 reviewing cases of pulmonary embolism presenting with abdominal pain identified 31 reported cases. Among the 26 patients who described the pain location, 83.9% experienced pain in the upper abdomen. In 22 cases with unilateral lung lesions, the abdominal pain corresponded to the side of the lung involvement in 95.5% of cases, except for one case with epigastric pain. In contrast, abdominal pain location was variable in patients with bilateral lung lesions.^[13]^ Flank pain secondary to PE is far rarer, and there are few reported cases that highlight this atypicality. *Amesquita et al* reported 3 cases presented with flank pain.^[15]^ The 1st case described a 52-year-old man who presented with a nonproductive cough, chest pain, and flank pain. He was misdiagnosed with pneumonia and treated with antibiotics. The patient returned the next day with a deteriorated status, along with hemoptysis. CT was performed, and PE was diagnosed. This highlights that PE greatly mimics pneumonia presentation. Similarly, our patient was initially treated as a case of pneumonia, and misdiagnosis most likely happened before the PE diagnosis. The 2nd case was for 2 postmenopausal women who presented with flank pain without chest pain. Similar to our case, one patient was evaluated by abdominal CT for renal pathologies. An incidental lower lobe infiltrate was noted, and a subsequent chest CT was performed to diagnose PE.^[13,15,16]^Hence, it’s advisable to extend the imaging toward the chest that while evaluating the abdominal pain by CT scan. Additionally, it should be kept in mind that PE can present with flank pain solely, adding more atypicality when chest pain is negative.

Additionally, a positive Murphy’s sign can be misleading.^[15–17]^ *Mansmann et al* reported a 42-year-old white male presented with typical cholecystitis. US was negative, and similar incidental findings on abdominal CT scan were noted.^[16]^

Abdominal pain in PE may result from hepatic or gallbladder distension due to right-sided heart failure, paradoxical embolism reducing abdominal organ perfusion, hypoxia-induced microemboli causing focal necrosis, or pulmonary hypertension leading to abdominal congestion. It can also arise from neurological effects like pseudoileus or as referred pain from diaphragmatic irritation and stimulation of thoracic sensory nerves. While thrombotic emboli are the most common cause of pulmonary embolism, other types of emboli exist, including fat, air, tumor, amniotic fluid, and septic emboli. The presentation of the case was not consistent with the aforementioned types. In particular, septic emboli occur when an infected focus showers emboli distally, such as an infected heart valve. The lack of a septic focus in this patient makes this type unlikely.^[13,18–20]^

The diagnostic approach to suspected acute PE starts with assessing hemodynamic stability, followed by estimating clinical probability using tools like the Wells, Modified Wells, or Geneva scores.^[21]^ High-probability cases proceed directly to CT pulmonary angiography (CTPA), while low- and intermediate-probability cases require D-dimer testing and possibly PERC criteria to rule out PE. Age-adjusted D-dimer thresholds improve specificity in older patients. CTPA is the preferred imaging modality, with V/Q scans, MRPA, or Doppler ultrasound as alternatives when contraindicated. Echocardiography can assist in unstable patients if thrombus or right heart strain is seen but is otherwise nonspecific. Emerging technologies, including AI-assisted imaging and advanced modalities like SPECT and dual-energy CT, are under investigation to enhance diagnostic accuracy.^[22,23]^

Initial management of acute PE focuses on supportive care, including oxygen for hypoxemia, cautious fluid resuscitation, and vasopressors for instability. Anticoagulation remains the mainstay, with LMWH or fondaparinux preferred, and initiation guided by hemodynamic status and clinical probability. Unstable patients require urgent imaging and often reperfusion therapy – systemic thrombolysis (preferred within 48 h), catheter-directed interventions, or surgical embolectomy if thrombolysis is contraindicated or unsuccessful. IVC filters are reserved for confirmed PE when anticoagulation is impossible or ineffective, with retrievable types preferred to reduce long-term DVT risk.^[24]^

Although this case has described a rare and clinically significant presentation, limitations include a lack of generalizability since it describes a single patient. In addition, although the resolution of the symptoms arising from both PE and flank pain suggests a direct causal relationship, the exact mechanisms by which this association exists are not fully understood and can’t be confirmed by reporting one case. These limitations underscore the necessity of reporting and studying further aspects of these atypical presentations.

## 4. Conclusion

This case highlights an exceptionally rare presentation of pulmonary embolism, with flank pain as the initial complaint. In the absence of identifiable abdominal pathology, clinicians should broaden their differential diagnosis to include PE, especially in the presence of thromboembolic risk factors. Early recognition and treatment remain critical in reducing morbidity and mortality. Awareness of such unusual presentations can help prevent misdiagnosis and ensure timely intervention in life-threatening conditions.

## Acknowledgments

We would like to thank the patient for participating in this study.

## Author contributions

**Conceptualization:** Mohammed Alra’e, Maaweya Jabareen, Hanan Qunibi.

**Data curation:** Mohammed Alra’e, Maaweya Jabareen, Hanan Qunibi.

**Project administration:** Mohammed Alra’e.

**Writing – original draft:** Mohammed Alra’e, Maaweya Jabareen, Saleh Juneidi.

**Writing – review & editing:** Mohammed Alra’e, Maaweya Jabareen.

**Resources:** Hanan Qunibi.

**Software:** Hanan Qunibi.

**Supervision:** Saleh Juneidi, Wasef Alhroub, Walid Tamimi, Musad Shawar.
